# Computer-aided assessment of the extra-cellular matrix during pancreatic carcinogenesis: a pilot study

**DOI:** 10.1186/s12967-019-1817-3

**Published:** 2019-02-28

**Authors:** Fabio Grizzi, Sirio Fiorino, Dorina Qehajaj, Adele Fornelli, Carlo Russo, Dario de Biase, Michele Masetti, Laura Mastrangelo, Matteo Zanello, Raffaele Lombardi, Andrea Domanico, Esterita Accogli, Andrea Tura, Leonardo Mirandola, Maurizio Chiriva-Internati, Robert S. Bresalier, Elio Jovine, Paolo Leandri, Luca Di Tommaso

**Affiliations:** 1 Department of Immunology and Inflammation, Humanitas Clinical and Research Center—IRCCS, Rozzano, Milan, Italy; 2 Humanitas University, Rozzano, Milan, Italy; 3 Internal Medicine Unit, Maggiore Hospital, Bologna, Italy; 4 Anatomic Pathology Service, Maggiore Hospital, Bologna, Italy; 5 “Michele Rodriguez” Foundation-Institute for Quantitative Measures in Medicine, Milan, Italy; 6 Department of Pharmacy and Biotechnology (FaBiT), University of Bologna, Bologna, Italy; 7 Surgery Unit, Maggiore Hospital, Bologna, Italy; 8 Ultrasound Center Internal Medicine A, Maggiore Hospital, Bologna, Italy; 9 CNR Institute of Neuroscience, Padua, Italy; 10 Department of Pathology, Humanitas Clinical and Research Center—IRCCS, Rozzano, Milano, Italy; 11 Kiromic Biopharma, Inc., Houston, TX USA; 12 Department of Gastroenterology, Hepatology & Nutrition, Division of Internal Medicine, The University of Texas MD Anderson Cancer, Houston, TX USA; 13 Histology Core, Humanitas Clinical and Research Center—IRCCS, Rozzano, Milan, Italy

**Keywords:** Pancreatic adenocarcinoma, Extra-cellular matrix, Degradation, Modeling, Fractals

## Abstract

**Background:**

A hallmark of pancreatic ductal adenocarcinoma is the desmoplastic reaction, but its impact on the tumor behavior remains controversial. Our aim was to introduce a computer -aided method to precisely quantify the amount of pancreatic collagenic extra-cellular matrix, its spatial distribution pattern, and the degradation process.

**Methods:**

A series of normal, inflammatory and neoplastic pancreatic ductal adenocarcinoma formalin-fixed and paraffin-embedded Sirius red stained sections were automatically digitized and analyzed using a computer-aided method.

**Results:**

We found a progressive increase of pancreatic collagenic extra-cellular matrix from normal to the inflammatory and pancreatic ductal adenocarcinoma. The two-dimensional fractal dimension showed a significant difference in the collagenic extra-cellular matrix spatial complexity between normal versus inflammatory and pancreatic ductal adenocarcinoma. A significant difference when comparing the number of cycles necessary to degrade the pancreatic collagenic extra-cellular matrix in normal versus inflammatory and pancreatic ductal adenocarcinoma was also found. The difference between inflammatory and pancreatic ductal adenocarcinoma was also significant. Furthermore, the mean velocity of collagenic extra-cellular matrix degradation was found to be faster in inflammatory and pancreatic ductal adenocarcinoma than in normal.

**Conclusion:**

These findings demonstrate that inflammatory and pancreatic ductal adenocarcinomas are characterized by an increased amount of pancreatic collagenic extra-cellular matrix and by changes in their spatial complexity and degradation. Our study defines new features about the pancreatic collagenic extra-cellular matrix, and represents a basis for further investigations into the clinical behavior of pancreatic ductal adenocarcinoma and the development of therapeutic strategies.

## Background

Pancreatic ductal adenocarcinoma (PDAC) represents the seventh leading cause of cancer-related death in the world, with an overall 5-year survival rate of 5%. Even localized disease has a 5-year survival rate of only 20%–25% [1]. In contrast to declining trends for other major cancers, death rates are rising in both sexes for PDAC [2]. This malignancy is often diagnosed in an advanced stage, leaving only palliative treatment options [3]. A growing number of studies has considerably improved our knowledge concerning the genetic and epigenetic alterations, the molecular perturbances and the precursor lesions, associated with the onset and the development of this malignancy [4–6]. In particular, changes in telomere length, complex karyotypes and multiple copy number alterations often spanning very large genomic regions, as well as several DNA changes have been reported [3, 7]. The latter consists of activating mutations in oncogenes, such as *KRAS*, or inactivating alterations in tumor suppressor genes, including *P16*, *TP53*, and *SMAD4* or in additional genes, such as *MLL3*, *TGFBR2*, *ARID1A*, *CDKN2a* and *ATM* [8]. These mutations, observed in non-invasive precursor lesions known as pancreatic intraepithelial neoplasia (PanIN) [9, 10], accumulate and drive neoplastic transformation and tumor progression [9, 11, 12]. However, despite these apparently encouraging results, this type of approach has produced no significant impact on the prognosis of PDAC [13]. Therefore, novel pathogenetic models are needed to explain the aggressive biological behavior, and dismal outcome of this malignancy as well as to suggest new and more effective options for its diagnosis and therapy [14]. Accumulating data indicate that not only alterations in malignant epithelial cells, but also extracellular matrix, surrounding cancerous cells play a critical, dynamic and cooperative role in the development of inflammatory as well as cancerous lesions [15, 16]. Histologically, PDAC is a complex structure, composed of infiltrating neoplastic glands embedded in an intense desmoplastic reaction. The latter consists of an ECM, non-neoplastic activated fibroblasts [17], myofibroblasts, cells of the immune system [18, 19], blood and lymphatic vessels. ECM includes collagens, non-collagen glycoproteins, glycosaminoglycans, growth factors and proteoglycans as well as modulators of the cell matrix interaction such as periostin, tenascin C, SPARC (secreted protein acidic and rich in cysteine) and thrombospondin [20, 21]. This framework represents the bulk of the cancer mass [22]. Interactions between the neoplastic and non-neoplastic cells and ECM have been proposed to stimulate the extensive desmoplastic reaction [23–26]. Although a critical role of stroma in pancreatic carcinogenesis had been recognized for many years, only recently the study of this crucial tissue component has gained a considerable interest and has been considered in clinical practice [14, 27].

There is accumulating evidence that while natural stroma can delay or prevent tumorigenesis, abnormal ECM components can promote tumor growth, and that this explains the low therapeutic response of pancreatic cancer patients [20, 28]. The complex interplay between tumor cells, non-tumoral cells and their ECM products also leads to dynamical changes in the transcriptional program of the cellular components, such as activated fibroblasts, stellate cells and inflammatory cells, which in turn promotes cancer cell motility, resistance to hypoxia and stromal neo-vascularity [29]. To date, mechanisms involved in the initiation and progression of these events are not completely understood. Chronic inflammation exerts a considerable impact in carcinogenesis, by inducing the deposition of a modified ECM tissue with qualitatively and quantitatively altered proteins in comparison with those detectable in normal pancreas. Such a condition is characterized by the progressive development of elevated tensional resistance stresses and high compression forces, both in intracellular and in extracellular compartments, and is associated with the perturbation of homeostasis [30]. This process causes progressive changes in tissue architecture and spatial organization of this organ and induces an increase in its stiffness. Tissue stiffness is now recognized as a risk factor for cancer development not only in pancreas, but also in other organs [31, 32]. However, although these modifications of pancreatic tissue structure have been qualitatively described and increased stromal collagen content has been reported in PDAC [33, 34], only a few studies have been focused to the quantitative assessment of the structure and the organization of stroma in this malignancy [35, 36].

It is now recognized that computer models are crucial for scientific procedures, and the modeling process represents a hypothetical-deductive approach in science [37, 38]. We performed this study with the aim to validate a novel method to quantify extra-cellular matrix deposition, geometrical spatial complexity and ECM degradation in histologic specimens from normal pancreatic tissue (nPA), inflammatory status (iPA) and PDAC. We used an automatically computer-aided image analysis system, which recognizes Sirius-red stained collagen fibers, quantifies the amount of pancreatic collagenic extra-cellular matrix (ECM) and its pattern, and simulates its degradation process. This novel methodology expands our knowledge of the ECM spatial organization and disposition in PDAC and may contribute knowledge relevant to clinically important aspects of this disease.

## Methods

### Patients

Formalin-fixed and paraffin-embedded specimens were obtained from 7 patients with diagnosed PDAC (3 males and 4 females, age ranged from 62 to 78 years) and 6 subjects (3 males and 3 females, age ranged from 56 to 74 years) with chronic pancreatitis, who had previously undergone surgical resection for neoplasm or non-malignant diseases respectively (10 images for case at 20× objective). As controls, pancreatic tissue specimens with no evident pathology (again 10 images for case at 20× objective) were obtained by autopsy from 5 individuals (3 males and 2 females, age ranged from 36 to 76 years). Patients’ clinical characteristics are reported in Table 1. Patients who underwent surgical procedure provided a written consent to the study participation.

**Table 1:** Clinical characteristics of patients included in the study

Patients	18
Sex	Male: 9 (50%)
	Female: 9 (50%)
Age (years)	65.8 ± 2.55 (range 36–78)
Histological diagnosis	PDAC: 7
	CP: 6
	NP: 5
Ca 19–9 (U/ml)	61.3 ± 24.4 (range 2.5–182)
CEA (ng/ml)	5.9 ± 2.35 (range 1.2–18.3)

*PDAC* pancreatic ductal adenocarcinoma, *CP* chronic pancreatitis, *NP* normal pancreas, *SE* standard error

### Histochemistry

Two consecutive 2-μm-thick sections were cut from each formalin-fixed, paraffin-embedded specimen. One was subsequently stained with haematoxylin & eosin solution, and the other was stained with a freshly prepared PicroSirius red collagen staining solution [39].

### Computer-aided morphometric and fractal analysis

**Fig. 1:**
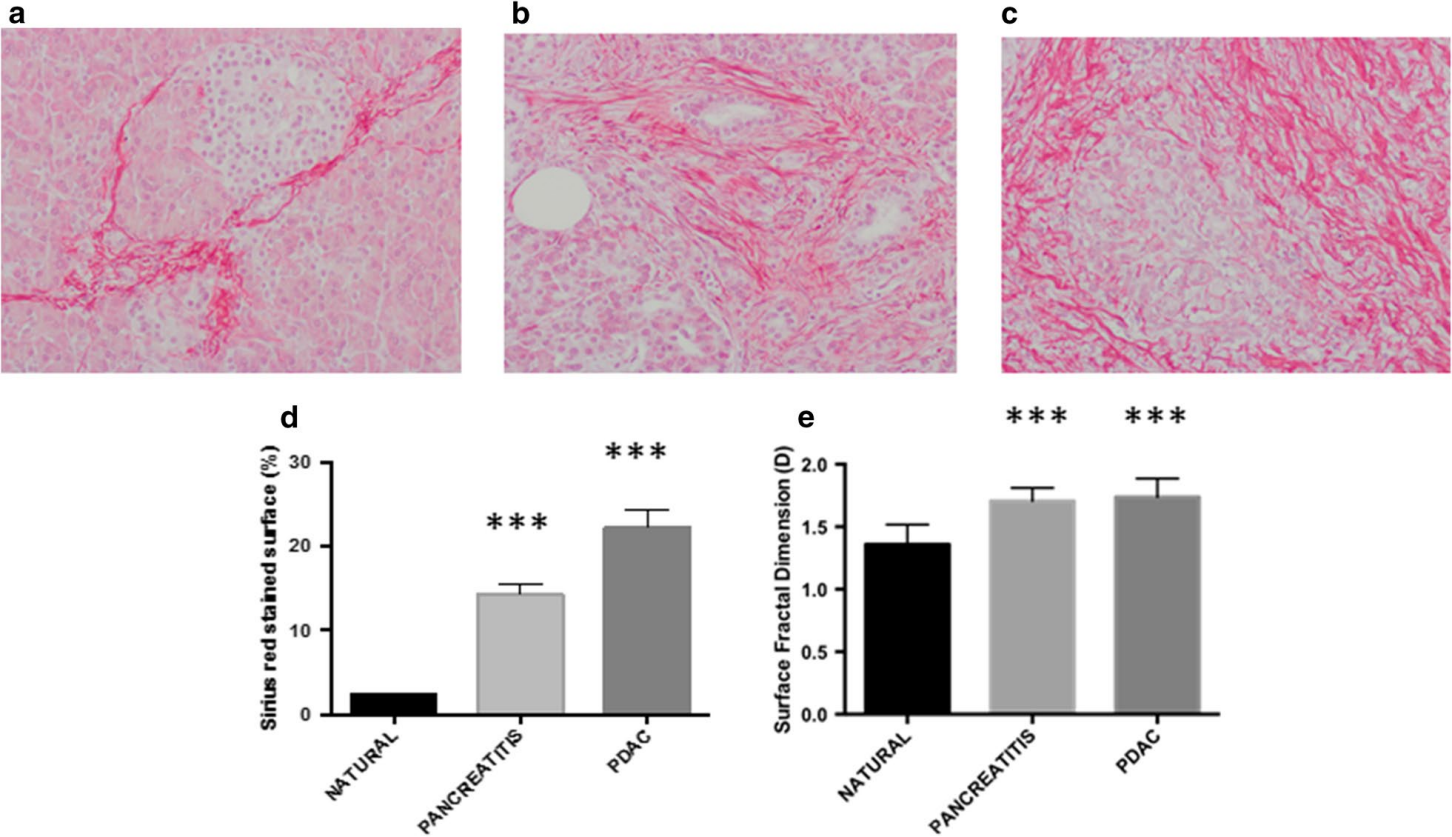
The deposition of collagen extracellular-matrix drastically increases from natural pancreas (**a**) to pancreatitis (**b**) and adenocarcinoma (**c**). We found statistically significant difference when comparing the percentage of ECM matrix (**d**) and its 2-D surface fractal dimension (**e**). ***p < 0.0001 by Student’s t-test
- *Sirius*-*red stained ECM surface* as the sum of all the areas of the collagen islets, expressed as a percentage of the pancreatic section surface area excluding any unfilled natural spaces and tissue-free spaces resulting from specimen manipulation.
- *2*-*D ECM fractal surface dimension* as a measure of its space-filling properties, which was automatically estimated by means of the box-counting method [40, 41] using the formula:
  $$D = \mathop {\lim }\limits_{\varepsilon \to 0} \inf \frac{{{ \log }\left( {N\left( \varepsilon \right)} \right)}}{{{ \log }\left( {1/\varepsilon } \right)}}$$
  where *D* is the box-counting fractal dimension, *ε* the side length of the box, and *N*(*ε*) the smallest number of boxes of side *ε* required to cover the complete surface of interest of the object (i.e. containing useful information). As the zero limit cannot be applied to biological objects, the dimensions were calculated as *D *=* d*, where d is the slope of the graph of *log* [*N*(*ε*)] against *log 1/ε*. The *log*–*log* graphs were plotted, the linear segments were identified using least squares regression, and their gradients were calculated using an iterative resistant line method, as previously described [42]. The concept of fractal dimension is shown in Fig. 2.

For each Sirius-red stained section, ten “regions of interest” (ROIs) were digitized at 20× objective magnifications by using an Olympus microscope (Olympus, Italy). Ad hoc software automatically selects collagen fibers based on RGB color segmentation. The same image intensity level was used throughout the study. The surface area of the fibrosis, its distribution variability and fractal dimension were automatically calculated for each digitized ROI. In brief, we geometrically defined the following:

- *Sirius*-*red stained ECM* as a set of irregularly shaped objects (collagen fragments or *islets*) that could be distinguished from the remaining tissue by their chemical affinity to Sirius red dye (Fig. 1a–c).

**Fig. 2:**
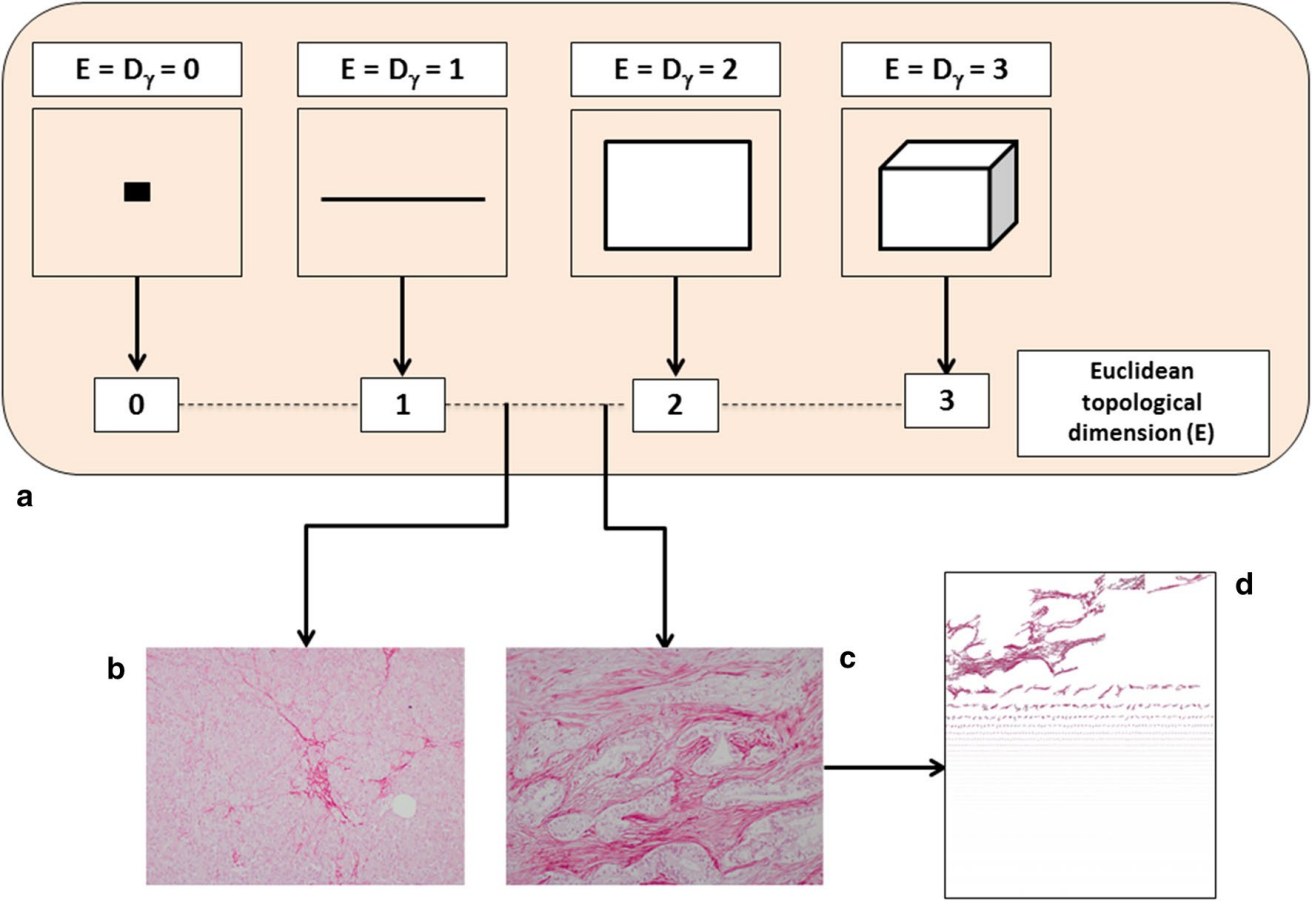
The fractal (i.e. non-integer) dimension is a real number that can be attributed to every natural object. The topological dimension of an object is indicated with the symbol Dγ, whereas the fractional dimension simply with D. For all Euclidean figures, Dγ and D are coincident, i.e., Dγ = D (**a**). This equality is not valid for the natural, including biological, objects. Natural objects can be roughly represented by Euclidean shapes (i.e., a tree resembles a cylinder, the sun is similar to a sphere, a mountain can be interpreted as a cone), but in reality, these shapes are not Euclidean figures. As suggested by Benoit Mandelbrot, it is possible to determine the Hausdorff–Besicovitch dimension or FD, of irregularly shaped objects through the covering procedure of the topological space of the object being measured. The software automatically estimates the 2D-fractal dimension of Sirius red stained pancreatic ECM (**b**, **c**). The more D tends to 2 the more the analyzed conformation tends to fill a 2D space and the greater it’s the geometrical complexity. Pancreatic desmoplasia consists of a set of irregularly shaped “collagen islets” (**d**)

### Computer-aided ECM degradation simulation

**Fig. 3:**
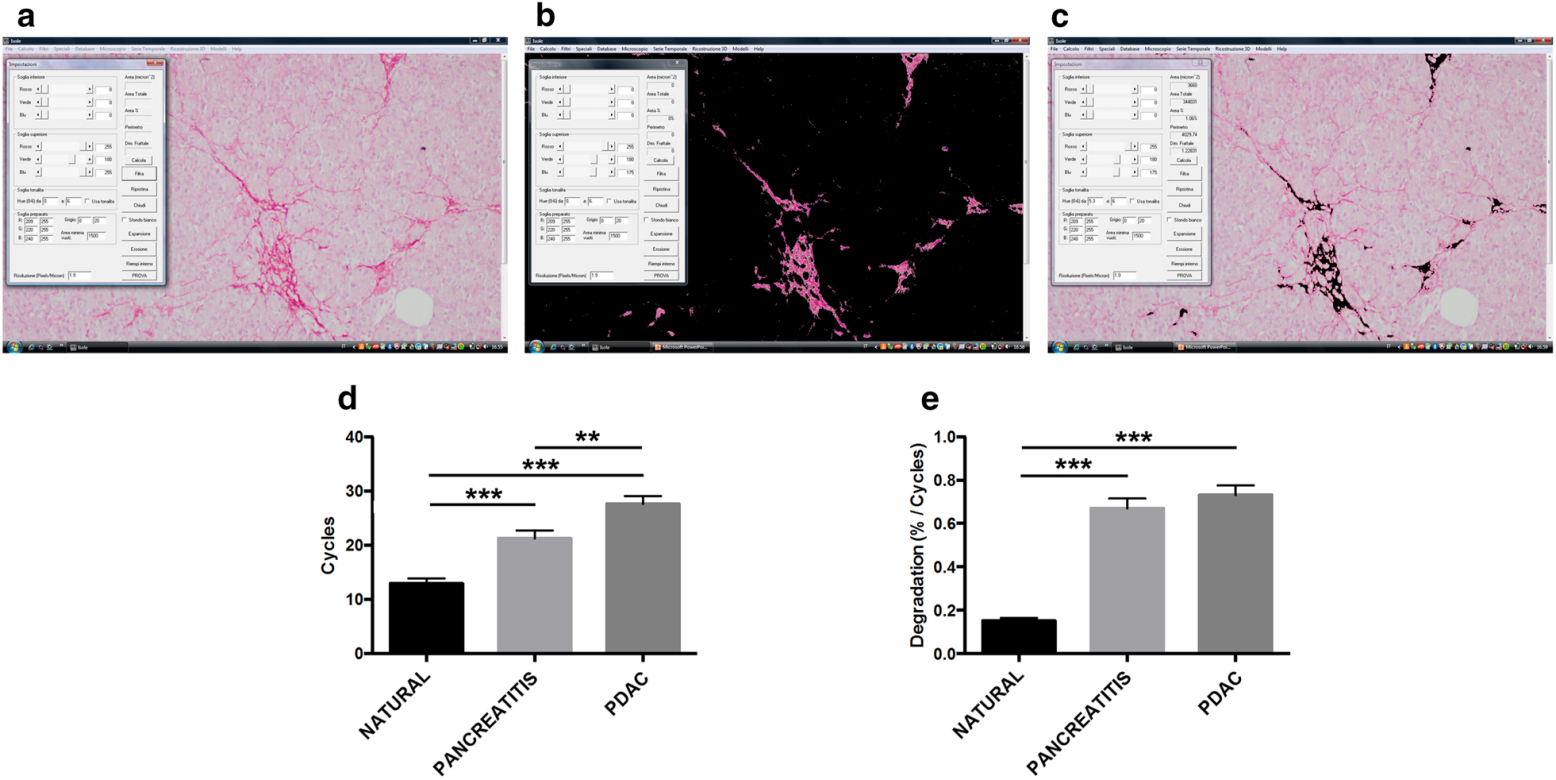
Computer-aided simulation of pancreatic ECM degradation (**a**–**c**). A statistically significant difference was found when compared the number of cycles necessary to erode the ECM in normal parenchyma versus parenchyma affected by pancreatitis and PDAC. A statistically significant difference was also found comparing the number of cycles **(d)** necessary to erode the ECM infiltrating the parenchyma affected by pancreatitis and that of PDAC. Also, when we analyzed the velocity of degradation (**e**) we found that this simulated phenomenon is faster in pancreatitis and PDAC than in natural pancreatic tissue. In contrast, no significant statistically differences were found when compared the simulated velocity of degradation between pancreatitis and PDAC (**e**). ***p < 0.001 by Student’s t-test; **p < 0.01 by Student’s t-test

A model simulating the two-dimensional degradation of the Sirius-red stained irregularly shaped collagen fibers (i.e. collagen islets) was developed (Fig. 3a–c). The adopted mathematical operation is called “erosion” [43, 44]. Erosion (i.e. removes pixels on object boundaries) is one of two fundamental operations (the other being *dilation*, i.e. adds pixels to the boundaries of objects in an image) in morphological image processing from which all other morphological operations are based. This mathematical function is dependent on the shape of the object. In other words the process of erosion is dependent on the degree of irregularity of the object. For any temporal cycle, the function eliminates the isolated pixels from the background and erodes the boundaries of the collagen fibers. As the ECM consists of a set of irregularly shaped fibers [45–50] with different size, the dynamical process of erosion depends on the fibers shape, size and spatial pattern [51]. At any cycle, the model automatically evaluated the “*Sirius*-*red stained ECM surface”* as the sum of the areas of the collagen islets, expressed in percentage. The “number of cycles” is defined as the time necessary to obtain a Sirius-red stained ECM surface equal to 0%. The higher is the irregularity of the collagen fibers, the higher is the number of cycle necessary to complete the degradation process. Thus, such number of cycles is a marker of fibers shape irregularity.

In addition to the number of cycles necessary to completely erode the Sirius-red stained ECM, we also considered a second parameter, i.e., the “mean velocity of degradation” (i.e. the amount of ECM erased during each cycle) obtained dividing the initial ECM surface with the number of cycles.

### Statistical analysis

Data are expressed as mean ± standard deviation (unless otherwise specified), and were analyzed using Statistica software (StatSoft Inc., Tulsa, OK, USA) and GraphPad Prism software version 7.0 application (GraphPad Software, San Diego, CA, USA).

Data comparisons were performed by using the unpaired Student t-test. P values of less than 0.05 were considered statistically significant.

## Results

### Morphometric and fractal analysis

In keeping with previous studies, we found a statistically significant progressive increase of collagen extra-cellular matrix deposition from normal pancreatic tissue (nPA) to inflammatory status (iPA) to pancreatic ductal adenocarcinoma PDAC [nPA: 2.23 ± 0.28%, iPA: 14.27 ± 1.27%, PDAC: 22.30 ± 2.031%; p < 0.0001] (Fig. 1d). With regard to the 2-D fractal surface dimension (i.e. space-filling property of a set of irregularly shaped ECM fibers) we found a statistically significant difference (p < 0.0001) in the geometrical spatial complexity of ECM between nPA (1.35 ± 0.02) versus iPA (1.70 ± 0.01) and PDAC (1.73 ± 0.01), but no differences were found between iPA and PDAC (Fig. 1e).

### Degradation simulation

As shown in Fig. 3d, we found a statistically significant difference (p < 0.001) when comparing the number of cycles necessary to erode the Sirius-red stained ECM in nPA (12.94 ± 0.94) versus iPA (21.27 ± 1.43) and PDAC (27.61 ± 1.43). Difference between iPA and PDAC was also significant (p < 0.003). In addition, when we analyzed the mean velocity of Sirius-red stained ECM degradation (Fig. 3e) we found that this process is faster in iPA and PDAC than in nPA and that the difference was statistically significant (p < 0.001). In contrast, no significant differences were found when comparing the simulated velocity of degradation between pancreatitis and PDAC (Fig. 3e). Table 2 reports all the data obtained by analyzing the geometrical features of Sirius-red stained ECM.

**Table 2:** Geometrical features of Sirius red stained pancreatic collagenic ECM and simulated degradation

	Computer-aided morphometric analysis				Computer modeling	
	Cases	ROI	Sirius-red stained surface (%)	Sirius-red stained collagenic ECM geometrical complexity (D)	Erosion cycles (n)	Erosion velocity
Natural pancreatic tissue	5	50	2.23 ± 0.28	1.36 ± 0.02	12.94 ± 0.94	0.15 ± 0.01
Pancreatitis	6	60	14.27 ± 1.27	1.70 ± 0.01	21.27 ± 1.43	0.67 ± 0.04
PDAC	7	70	22.30 ± 2.03	1.74 ± 0.01	27.61 ± 1.43	0.73 ± 0.04

Data are expressed as mean ± standard deviation

*ROI* region of interest, *PDAC* pancreatic adenocarcinoma

## Discussion

Pancreatic cancer is characterized by the formation of a dense, “desmoplastic” stroma (Fig. 1) [52, 53]. Whether this stroma drives the progression of PDAC or acts as a defense [54–56], still remains controversial. It has been shown that high stromal activity, as assessed by α-smooth muscle actin (α-SMA) expression, is associated with a poor prognosis in patients with PDAC [57]. Similarly, the high expression of ECM proteins such as SPARC [58] and periostin [59] is associated with a poor prognosis. Recently, Whatcott et al. observed a significant negative correlation between patient survival and ECM deposition in primary tumors [60].

Pancreatic fibrogenesis remains one of the most complex biological phenomena [61]. It is a dynamic process that is discontinuous in space and time, but advances through qualitatively different states. The non-linear progression of these states generates a complex structure that irregularly fills the surrounding environment (Fig. 2).

Several methods have been proposed to histologically quantify the stromal reaction in pancreatic carcinogenesis [62]. However, they have a number of substantial limitations, mainly due to the complex biology characterizing pancreatic ECM, and the highly irregular geometry that the stromal network assumes in real space, which cannot be quantified using the principles of Euclidean geometry (only capable of interpreting regular and smooth objects). The main feature of the newly generated ECM is the structural diversity of the Sirius-red collagen islets, shapes, sizes and distribution pattern [45–50]. Quantitative descriptors of ECM geometrical complexity can be usefully abstracted from the fractal geometry [45–50]. In general terms, fractal objects are mainly characterized by four properties: (a) the irregularity of their shape; (b) the self-similarity of their structure; (c) their non-integer or fractal dimension; and (d) *scaling*, which means that the measured properties depend on the scale at which they are measured. An object is geometrically self-similar when every smaller piece of the object is an exact, or nearly exact, duplicate of the whole object. Statistical self-similarity concerns biological objects, including many anatomic forms [63].

Here, we have developed an innovative computer-aided methodology to investigate some geometrical features of the stroma in chronic pancreatitis and PDAC. To our knowledge, this is the first study where advanced mathematical modeling techniques, such as algorithms for fractal and degradation assessment, were used for the study of the pancreatic tissue.

Modeling is the process of generating mathematical models. A scientific model can provide a way to read elements easily, which have been broken down to a simpler form. A model is a simplified representation of a system at some particular point in time or space intended to promote understanding of the real system. A simulation is the manipulation of a model in such a way that it operates in time or space to compress it, thus enabling one to perceive the interactions that would not otherwise be apparent because of their separation in time or space. A simulation is the implementation of a model. In other words, simulation is the imitation of the operation of a real-world process or system over time while the model represents the system itself, whereas the simulation represents the operation of the system over time. In line with previous studies [33–36], our findings suggest a significant higher amount of collagen fibers deposition in inflammatory and neoplastic pancreatic tissue in comparison with natural pancreas. Furthermore, the fractal analysis disclosed that collagen fibers in inflammatory and neoplastic tissue show a more irregular surface as compared to those seen in normal tissue (Fig. 1e). These data support the hypothesis that during the inflammatory and neoplastic states involving the pancreas a significant modification of stromal components occurs either in terms of total amount and spatial organization. Although our studies provides no direct assessment of tissue stiffness in chronic pancreatitis and in PDAC, it is conceivable to suppose that the deposited ECM leads to increase in both tissue solid stress and tissue interstitial fluid pressures, both of which may mediate vascular compression and dysfunction [64–66]. These changes may also explain the scarce drug penetration observed in PDAC tissue [64].

In addition to the fractal analyses, to investigate the dynamics of collagenic ECM degradation we applied the morphological “erosion” function (Fig. 3a–c). Our model revealed for the first time a statistically significant difference when compared the number of cycles (i.e. time) necessary to erode the ECM: lower in nPA and higher in iPA and PDAC (Fig. 3d). This finding might reflect not only the increased amount of ECM observed in iPA and PDAC, but also the collagenic ECM conformation. Additionally, when we analyzed the velocity of degradation (i.e. the amount of ECM erased during each cycle) we found that this phenomenon is faster in iPA and PDAC while no differences were found between pancreatitis and PDAC (Fig. 3e). This finding means a presence of more compact, and thus difficult to erode, collagen in neoplastic and inflammatory conditions.

Of note, our methodology may have future applications of direct clinical relevance. Indeed, we hypothesize that fractal analyses may be applied in an in vivo context, for improved geometrical and morphological analyses of pancreatic images from radiological examinations. Such as approach appears plausible, as fractal analysis have been already applied for the study of radiological images [67], though this has never been attempted for the study of the pancreas.

## Conclusions

The results generated by our automatic computer methodology suggest that three variables are important in the quantitative evaluation of pancreatic desmoplasia: (a) the size, (b) the shape, and (c) the pattern of arrangement of collagen fibers. In view of these preliminary results, it may be hypothesized that the complex modifications of the ECM conformation in addition to the changes of the ECM composition might promote an increase in pancreatic tissue stiffness. This condition is associated with an increased risk of cancer development. Despite the low number of enrolled subjects and the absence of direct assessment of the relationship between extra-cellular matrix deposition, ECM degradation simulation and pancreatic tissue stiffness, this study represents a pilot study for further well-designed and adequately-sized studies to confirm these preliminary but promising results. It is however indubitable that viewing pancreatic cancer as a system that is dynamically complex in time and space will probably reveal more about its underlying behavioral characteristics. This way of thinking may further help to clarify concepts, interpret new and old experimental data, indicate alternative experiments and categorize the acquired knowledge on the basis of the similitude and/or shared behaviors of very different tumors. It is encouraging that mathematics, theoretics, biology and medicine continue to contribute together towards a common quantitative understanding of cancer complexity.

## Abbreviations

PDAC: pancreatic ductal adenocarcinoma
pcECM: pancreatic collagenic extra-cellular matrix
nPA: pancreatic natural
iPA: pancreatic inflammatory
ROI: regions of interest
SPARC: secreted protein acidic and rich in cysteine
